# Smartphone-based intervention for postpartum depressive symptoms (Smart-e-Moms): study protocol for a randomized controlled trial

**DOI:** 10.1186/s13063-024-08304-5

**Published:** 2024-07-10

**Authors:** Daria Daehn, Caroline Meyer, Viola Loew, Jessica Wabiszczewicz, Steffi Pohl, Maria Böttche, Silke Pawils, Babette Renneberg

**Affiliations:** 1 Department of Education and Psychology, Freie Universität Berlin, Berlin, Germany; 2 Department of Medical Psychology, University Medical Center Hamburg‑Eppendorf, Hamburg, Germany

**Keywords:** PPD, Baby blues, Peripartum mental health, IBI, CBT, Prevention, Smartphone-delivered

## Abstract

**Background:**

Postpartum depression constitutes a significant public health issue, with prevalence rates ranging between 8 and 19% in high-income nations. Nevertheless, numerous barriers, including time constraints, societal stigmatization, and feelings of shame, contribute to the limited utilization of healthcare services during the postpartum period. Digital interventions offer an opportunity to enhance care for women experiencing postpartum depressive symptoms.

**Methods:**

We will conduct a two-arm randomized controlled trial to assess the effectiveness of a smartphone-based intervention in comparison to a treatment-as-usual control group in Germany. Our aim is to randomize 556 participants in a 1:1 ratio. Participants in the intervention group will be provided access to a preventive smartphone-based intervention called “Smart-e-Moms,” which incorporates therapeutic support and comprises 10 concise modules rooted in cognitive-behavioral therapy. For the intervention group, evaluations will take place at baseline (t0), prior to sessions 4 and 8 (intermediate assessments), and upon completing the intervention 6 weeks after baseline (t1). The control group’s assessments will be at baseline (t0) and 6 weeks after baseline. Follow-up assessments are scheduled at 12 and 24 weeks from baseline to examine the short-term stability of any observed effects. We anticipate that participants in the intervention group will exhibit improvements in their postpartum depressive symptoms (as measured with the Edinburgh Postnatal Depression Scale). Additionally, we will analyze secondary outcomes, including maternal bonding, stress levels, self-efficacy, satisfaction with the intervention, and healthcare utilization.

**Discussion:**

If Smart-e-Moms proves to be effective, it has the potential to play a significant role in postpartum depression care within German-speaking regions. Ideally, this intervention could not only benefit maternal well-being but also improve the prospects for healthy child development.

**Trial registration:**

German clinical trials registry DRKS00032324. Registered on January 26, 2024.

## Administrative information

Note: the numbers in curly brackets in this protocol refer to SPIRIT checklist item numbers. The order of the items has been modified to group similar items (see http://www.equator-network.org/reporting-guidelines/spirit-2013-statement-defining-standard-protocol-items-for-clinical-trials/).

**Table:** 

Title {1}	Smartphone-based intervention for postpartum depressive symptoms (Smart-e-Moms): study protocol for a randomized controlled trial
Trial registration {2a and 2b}	The trial is registered at the German clinical trials registry DRKS: DRKS00032324. The trial was registered on 26.01.2024.
Protocol version {3}	Version 1 of 27.03.2024
Funding {4}	This study is funded by the Innovation Committee (Innovationsausschuss) of the Joint Federal Committee (Gemeinsamer Bundesausschuss, GBA, no: 01VSF22024)
Author details {5a}	Daehn, Daria, MSc, Department of Education and Psychology, Freie Universität Berlin, Berlin, Germany, d.daehn@fu-berlin.de Meyer, Caroline, MSc, Department of Education and Psychology, Freie Universität Berlin, Berlin, Germany, caroline.meyer@fu-berlin.de Loew, Viola, MSc, Department of Medical Psychology, University Medical Center Hamburg‑Eppendorf, Hamburg, Germany, v.loew@uke.de Wabiszczewicz, Jessica, MSc, Department of Education and Psychology, Freie Universität Berlin, Berlin, Germany, j.wabiszczewicz@fu-berlin.de Pohl, Steffi, PhD, Department of Education and Psychology, Freie Universität Berlin, Berlin, Germany, steffi.pohl@fu-berlin.de Böttche, Maria, PhD, Department of Education and Psychology, Freie Universität Berlin, Berlin, Germany, maria.boettche@fu-berlin.de Pawils, Silke, PhD, Department of Medical Psychology, University Medical Center Hamburg‑Eppendorf, Hamburg, Germany, s.pawils@uke.de Renneberg, Babette, PhD, Department of Education and Psychology, Freie Universität Berlin, Berlin, Germany, b.renneberg@fu-berlin.de
Name and contact information for the trial sponsor {5b}	Gemeinsamer Bundesausschuss, GBA, no: 01VSF22024
Role of sponsor {5c}	The sponsor is not involved in the design of the study, data collection, administration, analysis and interpretation, the writing of the report, or the decision to the submission of the report

## Introduction

### Background and rationale {6a}

Postpartum depression (PPD) is the most commonly observed mental health disorder in the postpartum period, with a prevalence of 8–19% of individuals in high-income countries, posing significant risks to both women and their infants [1–4]. PPD is a condition that can cause symptoms such as emotional numbness, depressed mood, ambivalent feelings towards the child, lack of energy, feelings of guilt or worthlessness, and even thoughts of self-harm. These symptoms can last for more than 2 weeks [5, 6].

PPD can have severe and long-lasting negative effects on the entire family if left untreated [7]. It affects the psychological well-being of mothers, their overall quality of life, and their interactions with their infants, partners, and family members [8]. Furthermore, it increases the risk of recurrent depressive episodes [9]. Severe or chronic depression in mothers can also increase the risk of impaired child development. For example, PPD in mothers has been associated with an increased likelihood of behavioral problems and mental health problems in their children [9, 10].

Several treatment methods have been proven to be effective and feasible for women with PPD [2]. Among evidence-based treatment options, cognitive-behavioral therapy (CBT) has been shown to be the most effective (Branquinho et al., 2021b). However, despite the essential need for detection and treatment of PPD to prevent harmful consequences [8], the majority of women with PPD remain untreated [11]. The large discrepancy between the prevalence of PPD symptoms and treatment utilization is striking: only around 15% of women with PPD symptoms receive professional help, while healthcare utilization among those who have already tested positive for PPD is estimated at 22% [12]. Due to several barriers, women seem to be less likely to seek preventive care services and treatment for PPD in the postpartum period and therefore underutilize mental health services [13].

These barriers primarily include structural barriers, such as lack of time, inflexible scheduling, and difficulties with childcare [14, 15]. In addition, limited knowledge, societal stigma, and feelings of shame and guilt contribute to the low rates of detection and to the lack of treatment [13, 14, 16, 17 ]. Consequently, many women with PPD fail to access appropriate care despite experiencing significant distress and suffering. Additionally, there is a shortage of accessible prevention and care services for mothers with newborns [18].

To address these structural, societal, and individual barriers, internet-based interventions (IBIs) have emerged as a recognized and easily accessible means of providing specialized therapeutic care to women with the potential to both prevent and treat postpartum depressive symptoms [19–21]. IBIs targeting PPD commonly use evidence-based treatment elements, such as CBT techniques including behavioral activation and cognitive restructuring [22–24], psychoeducational components [24–26], and mindfulness and self-compassion practices [24, 27, 28].

Recent clinical trials have indicated that IBIs for women with PPD offer enhanced accessibility, improved treatment outcomes, and increased cost-effectiveness [28, 29]. IBIs offer anonymity, independence from location and time constraints, and can be tailored to individual needs [30, 31]. While international research supports the efficacy of IBIs for preventing and treating PPD, there is currently a lack of such digital care services specifically tailored for women in German-speaking countries [18]. In addition, many existing interventions are self-guided [22, 32]. However, research indicates that guided interventions have the potential to enhance effectiveness, adherence, and satisfaction with the intervention [33–35].

## Objectives {7}

We aim to develop a smartphone-based intervention for women with postpartum depressive symptoms and evaluate its effectiveness. The study focuses on mothers at risk for developing postpartum depression within the first 6 months after childbirth. The research questions addressed are as follows:

1. Does the therapist-guided smartphone-based intervention, Smart-e-Moms, reduce depressive symptoms more effectively than treatment as usual (TAU)?
2. Does the intervention result in lower stress, higher mother-infant bonding, and higher self-efficacy as compared to TAU?
3. How do users rate the usability, satisfaction, and utilization of the smartphone-based intervention?
4. Are there differences in healthcare service utilization between the intervention and TAU?

## Trial design {8}

We will conduct a parallel-group, two-arm randomized superiority trial, which compares the effectiveness of a smartphone-based intervention to a control group (treatment as usual, TAU) in Germany. This trial protocol is reported following the SPIRIT guidelines. The study has been registered in the German clinical trial register under https://www.drks.de/DRKS00032324. The protocol includes all items of the WHO Trial Registration Data Set. Participants will be randomly assigned to (a) the smartphone-based intervention (IG) or (b) treatment as usual (TAU). Randomization will follow a 1:1 allocation ratio, with sample size calculations suggesting a required sample of 556 (IG = 278; TAU = 278). For the intervention group (IG), assessments will take place at baseline (t0), before sessions 4 (t0_1) and 8 (t0_2) (intermediate measurements), and after completion of the intervention 6 weeks from baseline (t1). For the control group (TAU), assessments will take place at baseline (t0) and 6 weeks from baseline. Follow-up assessments will take place 12 weeks and 24 weeks from baseline to assess the short-term stability of any effects achieved.

## Methods: participants, interventions, and outcomes

### Study setting {9}

The study is jointly run by Freie Universität Berlin (FU Berlin) and Universitätsklinikum Hamburg-Eppendorf (UKE). FU Berlin will be responsible for data collection. Participants can download the DIRECT app on their personal smartphones and take part in the intervention and all assessments using the app. DIRECT is a digital research platform optimized for clinical psychology studies, which was developed at the Center of Mental Health and Digital Science of the FU Berlin and is continuously developed further. Referrals are made via social media, midwives, gynecologists, pediatricians, maternity hospitals, and participating health insurance providers.

### Eligibility criteria {10}

Inclusion criteria: Women between 6 weeks and 6 months after delivery with an elevated score of postpartum depressive symptomatology (EPDS ≥ 10, cutoff for clinically relevant depressive symptoms), without acute psychotic symptoms or acute suicidality may participate in the study after signing an informed consent form.

Exclusion criteria: No birth event in the last 6 months, acute psychotic and/or suicidal symptoms, insufficient knowledge of the German language, current psychotherapeutic treatment at the time of inclusion. There are no exclusion criteria regarding psychopharmacological treatment.

### Who will take informed consent? {26a}

Participating women will provide informed consent within the smartphone-delivered program.

### Additional consent provisions for collection and use of participant data and biological specimens {26b}

No biological specimens will be collected, and no secondary studies or analyses will utilize the data collected during the trial.

## Interventions

### Explanation for the choice of comparators {6b}

The control group can utilize standard care (treatment as usual, TAU) to determine the benefit of the intervention compared to routine care. However, due to the information provided during the study registration and by filling out the depression scale EPDS, it is assumed that the participants are better informed about PPD and their own vulnerability. Therefore, an informed standard care TAU is assumed. This group will be asked to provide information about well-being and maternal bonding at comparable time points. For both the intervention and control groups, participants are excluded if they have regular appointments with a psychotherapist at the time of inclusion. However, participants are allowed to reach out to any psychotherapeutic or psychiatric help available via routine care at their own discretion during the intervention. This approach was chosen to assess the additional benefit of the intervention as precisely as possible in a naturalistic setting.

### Intervention description {11a}

Smart-e-Moms is a therapist-guided smartphone-based intervention to reduce symptoms of postpartum depression in mothers. The intervention will be delivered on the DIRECT app developed by Freie Universität Berlin. At the beginning of the intervention, each participant will be assigned to a therapist who guides the participant through the intervention and provides written, individualized feedback at several time points. Smart-e-Moms consists of 10 sessions (see Table 1 for a description of the intervention components) that are delivered to the participants over the course of 5 weeks (i.e., 2 sessions/week). Every session lasts about 20 min and consists of psychoeducational content, interactive exercises, and summaries of the modules. The sessions are presented sequentially to the participants, with individualized feedback and encouragement given by the therapist after sessions 1, 3, 5, 7, 9, and 10. Additional information on topics related to postpartum mental health (i.e., depression and breastfeeding, child development) will be available throughout the intervention. Furthermore, throughout the intervention, participants will gain access to several additional tools, including a mood tracker, an activity planner, relaxation exercises, exercises for strengthening maternal bonding, and a pinboard to collect moments of happiness.

**Table 1:** Intervention components

Session number	Session name	Main topics	Key interventions	Session goal
1	Welcome session	Introduction of the intervention and individual goal setting, psychoeducation about PPD	Mood tracker^a^, expressive writing	Increasing the awareness of one’s own symptoms and normalizing negative emotions, establishing program motivation
Individualized written feedback provided by a psychologist				
2 + 3	Myths about motherhood	Myths about motherhood, association between thoughts and feelings	Collection of happy moments^a^, expressive writing	Debunking myths about motherhood through cognitive restructuring, reducing feelings of shame, increasing one’s focus on moments of happiness
Individualized written feedback provided by a psychologist				
4 + 5	Establishing soothing activities	Link between soothing activities and mood, dealing with challenges	Activity planner^a^, expressive writing	Behavioral activation and implementation of soothing activities—appropriate for the postpartum period—into everyday life with child
Individualized written feedback provided by a psychologist				
6 + 7	Stress management	Dealing with stress, psychoeducation on a mother’s need for self-care	Mindfulness and compassion-based exercises^a^, expressive writing	Dealing with stressful situations, integrating mindfulness practices and self-care into everyday life, increasing self-acceptance and self-compassion
Individualized written feedback provided by a psychologist				
8 + 9	Relationship to the child	Challenges in the mother–child relationship	Mindfulness exercises and playful activities for mother and child^a^, expressive writing	Normalizing ambivalent feelings, strengthening the mother–child relationship
Individualized written feedback provided by a psychologist				
10	Closing session	Summarizing key lessons learned from the intervention and introduction of strategies to maintain positive effects	Expressive writing	Maintaining positive changes, preventing relapse
Individualized written feedback provided by a psychologist				

^a^These functions are available to users throughout the intervention from the time of introduction

The unique aspect of Smart-e-Moms is its participatory development, ensuring that the app is specifically tailored to the needs of postpartum women. For instance, audio recordings of women sharing their experiences with PPD are integrated throughout the intervention to normalize users’ experiences and provide reassurance that they are not alone. Moreover, Smart-e-Moms offers continuous support from a psychologist, providing individualized feedback and encouragement throughout the intervention. The psychologists will be trained psychotherapists and/or psychotherapists in training working under the supervision of a trained and experienced psychotherapist. All participating clinicians will receive technical training and learn how to deliver individualized feedback to participants. In addition to the individualized guidance, Smart-e-Moms combines a comprehensive set of psychotherapeutic elements (e.g., CBT-tools, expressive writing, psychoeducation, mindfulness exercises), which distinguishes Smart-e-Moms from other German IBIs for perinatal women without guidance (Mind:Pregnany; [36]), or those focusing on informing about PPD [37]. Moreover, other German IBIs for perinatal women are not specifically tailored to the postpartum period (Mind:Pregnany,[36],I-PREGNO; [38]). A detailed overview of the intervention can be found in Table 1.

The therapists will be trained psychotherapists and/or psychotherapists in training working under the supervision of a qualified psychotherapist. All participating clinicians will receive technical training and learn how to deliver individualized feedback to participants. A detailed overview can be found in Table 1.

### Criteria for discontinuing or modifying allocated interventions {11b}

Participants can leave the study at any time for any reason if they wish to do so without any consequences.

Engagement in the program (i.e., starting and/or completion of sessions) will be automatically logged. After 1 week without starting or completing a new session, participants will receive a reminder message. Participants will still be invited to fill out all assessments even if they discontinue working on the sessions.

### Strategies to improve adherence to interventions {11c}

By nature, the intervention is highly manualized and standardized. All therapists will receive a manual and comprehensive training for using the app and delivering individualized feedback to the participants. To enhance the therapists’ training and ensure adherence of the participants, treatment will be closely monitored by a supervisor.

Engagement by the participants will be automatically logged and monitored by the therapists. Their weekly, individualized feedback messages are focused on encouraging the participants to continue working on the sessions. Participants receive push-up notifications in the app whenever individualized feedback by the therapist, a new session, or an assessment is available to them. To ensure engagement in the follow-up assessments, participants in both the intervention and the TAU group will receive monetary compensation with slightly higher compensations for the TAU group.

### Relevant concomitant care permitted or prohibited during the trial {11d}

For both the intervention and TAU groups, participants are excluded if they have regular appointments with a psychotherapist at the time of inclusion. However, participants are allowed to reach out to any psychotherapeutic or psychiatric help available via routine care during the intervention at their own discretion. We will assess medication use and additional health care utilization to analyze potential differences in the utilization of health services between the groups. This approach was chosen to assess the additional benefit of the intervention as precisely as possible in a naturalistic setting.

### Provisions for post-trial care {30}

N/a: There is no ancillary post-trial care or compensation. All participants in both the control group and the intervention group will be monitored throughout the whole follow-up period and can contact the study team. In case of crisis or suicidality, they will be offered a personal call by a trained psychotherapist.

### Outcomes {12}

#### Primary outcome measures

Edinburgh Postpartum Depression Scale (EPDS, [39]).

The primary outcome of the intervention will be the reduction of postpartum depressive symptoms, as measured by the Edinburgh Postpartum Depression Scale [39]. The self-report questionnaire consists of 10 items which are rated from 0 to 3 and summed to determine the overall score. A score of 9.5 or above is indicative of probable depression. The EPDS includes one item to assess suicidal ideation and self-harm tendencies that will be used to monitor potential self-harm throughout the intervention. The EPDS has demonstrated satisfactory validity and reliability and is the most commonly used outcome measure to assess postpartum depression. The German version of the EPDS has good psychometric properties [40]. The mean change from baseline will be compared between the intervention and the control group at post-assessment (t1), which is the most relevant time point of interest, and at follow-ups 1 and 2.

#### Secondary outcome measures

All secondary outcome measures have shown satisfactory reliability and validity in previous studies.

#### Postpartum Bonding Questionnaire [41]

Mother-infant bonding will be assessed with the Postpartum Bonding Questionnaire. It is a 16-item self-report scale with a rating from 0 to 5. The total score can be between 0 and 80, with a lower score indicating a better mother-infant bond. According to Brockington’s factor solution, 3 subscales are proposed: impaired bonding with a threshold of 12, rejection and anger with a threshold of 17, and threatened rejection with a range from 13 to 16. These scales showed an internal consistency of *α* = 0.85 of the German questionnaire. The mean change from baseline will be compared between the intervention and the control group at post-assessment (t1), which is the most relevant time point of interest, and at follow-ups 1 and 2.

#### General Self-Efficacy Scale [42]

This 10-item self-report scale assesses self-efficacy. Items are rated between 1 and 4 which results in an overall score between 10 and 40. Internal consistency varies in German samples between a Cronbach’s alpha between 0.89 und 0.94 [43]. The mean change from baseline will be compared between the intervention and the control group at post-assessment (t1), which is the most relevant time point of interest, and at follow-ups 1 and 2.

#### 1-Item Perkhofer Stress Scale [44]

Stress will be assessed with the 1-item Perkhofer Stress Scale, which is a combination of the Visual Analogue Scale and the Figure Rating Scale [44]. It consists of a horizontal line, with the left end representing no stress and the right end representing the greatest stress. Below the line, 6 emojis depict smiling faces at different levels. The scale shows satisfactory psychometric properties. The mean change from baseline will be compared between the intervention and the control group at post-assessment (t1), which is the most relevant time point of interest, and at follow-ups 1 and 2.

#### Questionnaire for the Assessment of Medical and non-Medical Resource Utilisation in Mental Disorders [45]

The FIMPsy can be used to assess the utilization of health care services in patients with mental disorders and thereby facilitate economic evaluations. We will use a selection of items and adapt them specifically for our target group.

The mean utilization score will be compared between the intervention and the control group at post-assessment (t1), which is the most relevant time point of interest, and at follow-ups 1 and 2.

#### Other measures

Other measures include sociodemographic, clinical, and pregnancy-related variables. Symptoms of birth-related post-traumatic stress disorder (PTSD) will be assessed with the City Birth Trauma Scale [46]. To record adverse childhood experiences the Childhood Trauma Screener [47] will be used.

The User Version of the Mobile Application Rating Scale (uMARS, [48]) will be used by end-users to assess the quality of mHealth apps. The uMARS consists of four objective quality subscales (engagement, functionality, aesthetics, information) and one subjective quality subscale (20 items in total). An additional subscale measures the perceived impact of the evaluated app (6 items). All items are measured on a 5-point scale. The uMARS showed excellent internal consistency (*α* = 0.90). Mean scores will be used to assess usability at post-assessment (t1).

Expectations before treatment will be assessed as a potential moderator with the Patient Questionnaire on Therapy Expectation and Evaluation [49] which consists of 11 items. Furthermore, we will use an IBI-adapted version of the 12-item Working Alliance Inventory [50] to assess the therapeutic alliance as a potential moderator.

### Participant timeline {13}

**Fig. 1:**
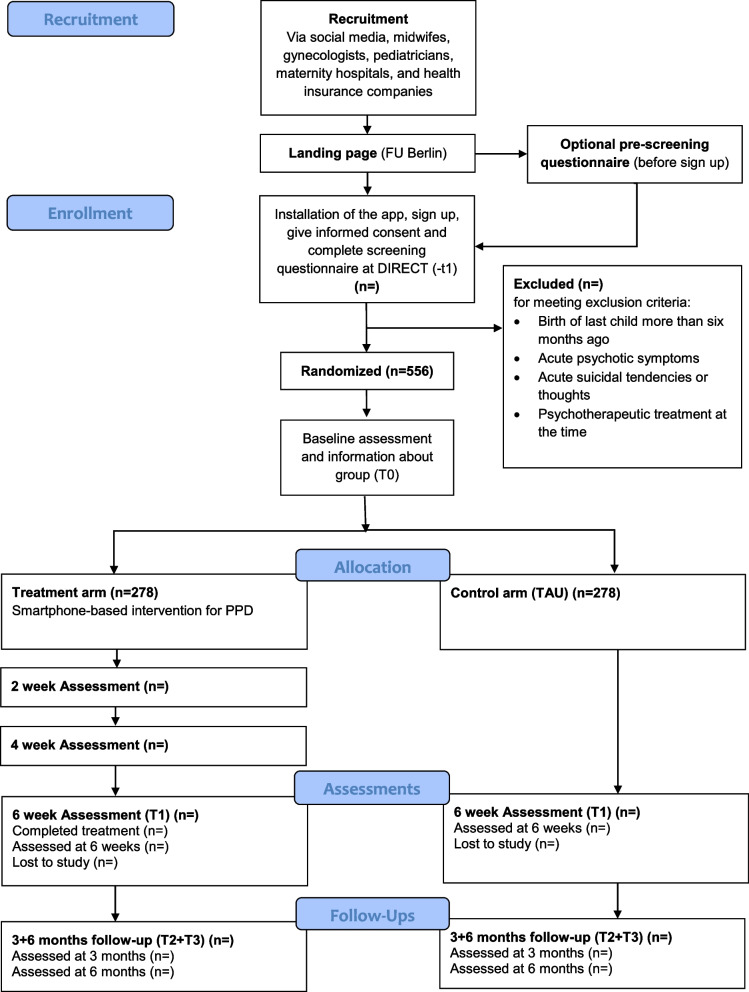
CONSORT flow diagram

Figure 1 depicts the CONSORT flow diagram. Potential participants are directed to a landing page that provides information about the trial. After downloading the DIRECT app and creating an account for the intervention, participants give electronic informed consent and provide their contact information (mail and phone number). Afterwards, participants fill out a screening questionnaire to make sure they fulfill the inclusion criteria. In case participants screen positive for suicidality or psychotic symptoms, a follow-up call will be made by a trained psychotherapist to assess eligibility using the corresponding modules of the Mini International Neuropsychiatric Interview [51] and the Structured Clinical Interview for DSM-5 [52]. Participants eligible for inclusion will complete the baseline assessment (t0) and are randomized to either the intervention group (IG) or the control group (TAU). Both groups receive invitations to fill out the assessments after the completion of the intervention (IG), or at 6 weeks (TAU) (t1), 12 weeks (t2), and 6 months (t3) after allocation.

### Sample size {14}

To ensure sufficient power for the effects at all time points, we considered potential dropout and power analysis for the last follow-up time point after 6 months (T3) using G*power. Dropout for patients with a depressive disorder in IBIs over the course of 6 months was estimated to be 50% based on previous literature [53]. For smartphone-based interventions targeting depressive symptoms, meta-analyses found small (*g* = 0.28, [54]) to small-to-moderate (*g* = 0.383, [55]) effect sizes when comparing apps to control conditions. As the TAU group in our study must be considered an informed TAU group due to the screening process, the calculation of our sample size is conservatively based on a small effect size (*d* = 0.3). The sample size calculation is conservatively based on a small effect size (*d* = 0.3), as the TAU group must be considered an informed TAU group due to the screening process. To find this effect in a randomized controlled trial with a one-tailed *t*-test (alpha error level = 5%, power = 0.80) for independent samples, *n* = 139 participants per treatment group (total *n* = 278 participants) are needed to complete the t3 assessment. Considering the dropout rate, a total sample of *N* = 556 participants is needed at the time of randomization (1:1 randomization: *n* = 278 participants in IG; *n* = 278 participants in TAU).

### Recruitment {15}

To ensure adequate participant enrollment, we will use several recruitment channels. (1) Social media channels will be used to provide information about the study through regular posts and stories, and ads will be placed on social media linking to the registration website. (2) Maternity clinics, midwives, gynecologists, and pediatricians will be recruited throughout Germany during the first year of the project to inform women about the intervention in routine care and to invite them to participate in the study. Pediatricians and midwives should be well acquainted with the offer and be able to provide information about it. For this, they receive an expense allowance from the project. The clinics should make women aware of the offer and inform them about it before birth or during the so-called U2 examination that routinely takes place at 3–10 days after birth in Germany. (3) Participating health insurance providers will inform women after birth as part of the routine information package that all women receive after the childbirth to draw attention to special services and offers provided by the respective health insurance provider.

## Assignment of interventions: allocation

### Sequence generation {16a}

A randomization list will be provided to the DIRECT software developers by a third party and entered into the system. There is no possibility for the stakeholders of the study to access/see this list. Participants meeting the inclusion criteria are randomly allocated to one of the groups by the trial staff via clicking a button in the admin panel. The randomization list is computer-generated using permuted block randomization using the “block rand” package in R. The sequence is generated by an external researcher and directly sent to the software developers who are adding the randomization list to the study.

### Concealment mechanism {16b}

After participants complete their baseline assessment, the coordinator is able to randomize the participant into intervention or TAU groups via a button on the admin panel without any knowledge of the order in the randomization list. The randomization list cannot be accessed or changed by the study team. After participants complete the baseline assessment, they are randomized by pressing a button in the study admin panel (i.e., a web application that is used by the study team to manage the study). This button assigns the participant to the next free seat on the randomization list. Since the randomization list is not accessible to the study team, it is not known what group a participant will be assigned to, prior to pressing the button.

### Implementation {16c}

Randomization will occur at the patient level. Participants enroll in the trial and undergo screening and baseline assessments. Once patients have consented to participate and provided baseline data, they will be randomized to either the intervention group or the TAU group in a 1:1 ratio. The study administrators will conduct the randomization in the DIRECT admin panel after the baseline assessment is completed.

## Assignment of interventions: blinding

### Who will be blinded {17a}

Due to the nature of psychotherapeutic intervention trials, neither participants nor therapists can be blinded to treatment allocation. Biostatisticians remain blinded to allocation.

### Procedure for unblinding if needed {17b}

N/a: As blinding is not possible due to the study design, unblinding will not be needed.

## Data collection and management

### Plans for assessment and collection of outcomes {18a}

Our study focuses on postpartum depressive symptoms as the primary outcome, with secondary measures including mother–child bonding, self-efficacy, stress, app quality, and healthcare utilization. Other measures encompass sociodemographic, clinical, and pregnancy-related variables. Outcomes will be measured by self-reported questionnaires, which are assessed at baseline (t0), after completion of the intervention 6 weeks after allocation (IG) or 6 weeks after allocation (TAU) (t1), and at 12 weeks (t2) and 6 months (t3) after allocation. Participants who receive the intervention will also complete an intermediate assessment before session 4 (t0_1) and session 8 (t0_2). All questionnaires will be presented via the DIRECT app that also hosts the intervention. A detailed overview of all assessments can be found in Table 2.

**Table 2:** Schedule of enrolment, interventions, and assessments

**Time point**		**Study period**							
		**Enrollment**	**Baseline**	**Allocation**	**Post-allocation**				
		(− t1)	(t0)	(0)	(t0_1) Session 4, ~ week 2	(t0_2) Session 8, ~ week 4	(t1) After completion, ~ week 6	(t2) Follow-up, week 12	(t3) Follow-up, week 24
**Enrolment**									
	Eligibility screening	X							
	Informed consent	X							
	Study suitability	X							
	Allocation			X					
**Interventions**									
	Smart-e-Moms				X	X	X	X	X
	TAU						X	X	X
**Assessments**	EPDS	X	X		X	X	X	X	X
	PBQ-16		X				X	X	X
	GSE		X				X	X	X
	Stress		X		X	X	X	X	X
	uMARS						X		
	App utilization						X		
	FIMPsy (adapted)		X				X	X	X
	Sociodemographic and clinical data		X						
	Birth-related data		X						
	City BiTS		X						
	CTS		X						
	PATHEV		X						
	WAI-SR				X	X	X		

*TAU* Treatment as usual, *EPDS* Edinburgh postnatal depression scale, *PBQ-16* Postpartum Bonding questionnaire, *GSE* Self-efficacy expectancy questionnaire, *Stress* Perkhofer stress scale, *uMARS* User version of the mobile application rating scale, *FIMPsy (adapted)* Questionnaire for the assessment of medical and non-medical resource utilisation in mental disorders, *City BiTS* City birth trauma scale, *CTS* Childhood trauma screener, *PATHEV* Patient questionnaire on therapy expectation and evaluation, *WAI-SR* Working alliance inventory—short revised

### Plans to promote participant retention and complete follow-up {18b}

Participants will receive push-up notifications that questionnaires are available in the app and will be reminded once if they do not complete the assessment. Participants who discontinue working on the sessions will still be invited to participate in the assessments. They will receive a monetary incentive to complete assessments. Participants in the intervention group will receive €15 for completing the 3-month follow-up assessment and an additional €25 for completing the 6-month follow-up assessment. Participants in the TAU group will receive €15 for completing the 6-week post-intervention assessment, an additional €15 for completing the 3-month follow-up assessment, and an additional €45 for completing the 6-month follow-up assessment.

### Data management {19}

All data is gathered online and will be recorded automatically by the DIRECT app. There are several measures in place to ensure data quality and safety. All questionnaires are tested beforehand to ensure correctness and completeness. The assessments are conducted automatically within the app, which ensures that questions are not skipped accidentally. Answers are synched with the server immediately or are stored locally for later synchronization in cases of internet disconnects. The data assessment and export features of the system have been tested in several studies and have proven to be reliable and robust. All admin personnel will receive technical training in working with the system to ensure all data collection processes are conducted and monitored according to the protocol.

Part of the anonymized data is made publicly available on an internet database for open access documentation. Before selecting the data, consultation with the data protection officer will take place.

### Confidentiality {27}

Participants will register on the DIRECT app with their e-mail address and telephone number to receive reminders, recover their accounts, and contact them in case of emergency. All data is stored on a secure server, and personal data will only be accessible to the supervising therapist and the study team. Personal data will be stored separately from questionnaire data to ensure data safety.

The data is saved locally on the smartphone device and is protected via the app password and any safety measurement the participants are using on their own device. The data is synchronized through encrypted (SSL) data transfer to a server (location: Germany). The server is provided by Hetzner GmbH, an ISO 27001 hosting provider that ensures compliance with the EU General Data Protection Regulation (GDPR). The data needs to be sent and stored on a server to ensure that clinical staff can support participants via the admin panel. Access to the admin panel is protected through two-factor authentication. Data export is only possible for study coordinators and again protected by two-factor authentication. The data export is pseudonymized by assigning an internal ID (hash). No personal data is exported. Study IDs and personal data are not matched outside of the admin panel. Data will be deleted upon request by participants (following GDPR regulations). Upon completion of the trial, all data will be exported and securely stored on an encrypted device for 10 years following good practice guidelines for clinical trials. This data will not contain personal information. After completion of the study with the follow-up assessments, all data from the app and the servers will be deleted.

### Plans for collection, laboratory evaluation, and storage of biological specimens for genetic or molecular analysis in this trial/future use {33}

N/a: No biological specimens will be collected.

## Statistical methods

### Statistical methods for primary and secondary outcomes {20a}

It is possible that some participants may not utilize the app, despite being assigned to the intervention group, and yet still participate in the surveys (non-compliance). We will deal with non-compliance in the analyses in two different ways. For the primary analyses, we will include data from all randomized patients and evaluate both (1) the effect of assignment to the intervention and (2) the effect of the intervention itself. For the estimation of the causal effect of the assignment to intervention on the primary and secondary endpoints (intention-to-treat analysis), the mean values between the two study groups at T1, T2, and T3 will be compared using *t*-tests and MANOVAs within the framework of structural equation models (SEM). For estimating the causal effect of the intervention itself on the primary and secondary endpoints, we treat all participants who were assigned to the intervention group but did not use the app (and did not drop out before the measurement of the first endpoint) as members of the control group (as-treated analysis). As this invalidates randomization, we will control for selection bias by adding the variables measured at baseline as covariates in the model. We assess all endpoints also at baseline, as this helps to assure the ignorability assumption (e.g., Cook, Steiner, & Pohl, 2010). Assumptions on distribution and homoscedasticity are tested and, if violated, relaxed (by using respective estimators or multi-group modeling).

To investigate the utilization of care services, means on individual items of the FIMPsy will be compared between the two treatment groups via *t*-tests in SEM.

For the intervention group, usability and acceptance of the intervention are analyzed using descriptive statistics to describe satisfaction (CSQ-i, SUS) and utilization behavior. The different recruitment channels for the intervention are surveyed and descriptively analyzed.

### Interim analyses {21b}

N/a: No interim analysis is planned in this trial.

### Methods for additional analyses (e.g., subgroup analyses) {20b}

#### Differential effects

We will evaluate whether the intervention effect differs between subgroups in a moderator analysis. For this, we will include the variables measured at baseline as further independent variables in the analysis model and evaluate their two-way interaction with the intervention variable on the endpoints (e.g., age, childbirth-related trauma, history of depressive episodes, number of children, income, subjective socioeconomic status, pregnancy complications, birth complications, mode of delivery, pregnancy intention, health care utilization, and level of education).

#### Extensity of app use

In additional analyses, we will explore which aspects are related to the extensity of app use. For this, we will only consider the data of the intervention group to explore in which way the number of modules taken related to the endpoints, we will regress the endpoint variables to the number of modules taken in a SEM framework. As the number of modules is not randomized, we will furthermore control for covariates (e.g., sickness, chronic illnesses of household members, working hours, number of children).

### Methods in analysis to handle protocol non-adherence and any statistical methods to handle missing data {20c}

#### Dropout

To deal with missing data due to dropout, analyses are conducted in the context of structural equation modeling and estimated by full information maximum likelihood using all variables measured at baseline as auxiliary variables.

#### Item-nonresponse

Missing values due to item-nonresponse are imputed via single imputation using (if possible) all other variables in the imputation model.

#### Protocol non-adherence

We handle protocol non-adherence in two ways: First, we estimate the causal effect of the assignment to the intervention on the endpoints (intention-to-treat analyses); second, we estimate the causal effect of the intervention on the endpoints by using the actual intervention taken (as-treated analyses) and accounting for selection bias via covariates in the analysis.

### Plans to give access to the full protocol, participant level data, and statistical code {31c}

Part of the anonymized data is made publicly available in an Open Science Framework (OSF) repository. Before selecting the data, consultation with the data protection officer will take place.

## Oversight and monitoring

### Composition of the coordinating center and trial steering committee {5d}

The RCT will be conducted at Freie Universität Berlin with day-to-day support: The study team meets weekly and is supervised by the principal investigator, who also supervises the trial. Monthly meetings take place with all consortium partners to discuss key milestones as well as the conduct of the research, ensuring the integrity of the protocol and conduct of the study. Any significant amendments to the study protocol will be provided to and approved by the ethics committee of Freie Universität Berlin before implementation.

There is no independent trial steering committee, but several advisory boards will be implemented with regular meetings for the entire duration of the project: A user advisory board will be implemented to represent the patients’ interests, a scientific advisory board will be implemented to provide guidance during development and evaluation of the intervention, and a sustainability advisory board will be implemented to develop a long-term implementation plan to make the app accessible for women after the end of the project free of charge.

### Composition of the data monitoring committee, its role and reporting structure {21a}

There is no data monitoring committee planned for this trial. All data will be automatically recorded via the DIRECT app.

### Adverse event reporting and harms {22}

Previous studies on the implementation of online interventions indicate robust treatment effects. We, therefore, expect a significant improvement in depressive symptoms. The risk of worsening through treatment is about 6% in internet-based therapies [56]. Participants with a particularly high risk of self-harm or harm to others (serious suicidal thoughts, current psychotic symptoms) will be excluded from participation by pre-screening according to defined exclusion criteria (see Sect. 10.3). Prospective study participants at risk of suicide and those who show evidence of psychotic symptoms will not be included in the study. However, they will be contacted by telephone and given information about where they can find help. If the telephone call reveals indications of current danger to self or others, further measures for support are initiated in consultation with the participating person, if necessary. One of the regularly used questionnaires (EPDS) directly contains an item on suicidality (“In the last 7 days or in the days since birth, the thought of harming myself came over me”). If the self-report shows indications of self-harm, the participants are called promptly (during the day Monday–Friday within 24 h) by supervisors (specific to the project), an emergency plan will be developed, and further measures are taken if necessary. In the app, emergency numbers and contact points are also available and easily accessible at all times.

There are no plans to collect unexpected harm systematically. However, participants will be asked to report any problems or adverse events resulting from the intervention in an open format at the end of every other session to monitor unexpected harm.

### Frequency and plans for auditing trial conduct {23}

There will be no independent audits for this trial. Monthly meetings take place with all consortium partners to discuss key milestones as well as the conduct of the research, ensuring the integrity of the protocol and conduct of the study.

### Plans for communicating important protocol amendments to relevant parties (e.g., trial participants, ethical committees) {25}

Any amendments to the study protocol have to be approved by the ethics committee of Freie Universität Berlin. Approved changes to the protocol will be reported in the trial register and in the primary publication about the trial.

## Dissemination plans {31a}

The trial results will be published in peer-reviewed journals and presented at scientific conferences.

## Discussion

To the best of our knowledge, this is the first RCT to examine the effectiveness of a smartphone-based therapist-guided intervention for German-speaking women with postpartum depressive symptoms. This intervention incorporates common CBT elements, such as behavioral activation and cognitive restructuring, psychoeducational elements, and elements of mindfulness and self-compassion. However, the content was adapted to the specific situation after birth. In 10 guided sessions, Smart-e-Moms addresses myths about motherhood, establishing soothing activities, stress management, and strengthening the mother–child relationship.

If the effectiveness of the intervention is demonstrated, Smart-e-Moms has the potential to circumvent many of the barriers to conventional healthcare utilization after childbirth. For instance, it offers women the possibility to flexibly decide where and when to work with the intervention. In addition, it is also suitable for women who would otherwise not seek conventional treatment due to feelings of shame. Smart-e-Moms has thus the potential to be an easily accessible, low-threshold intervention that offers anonymity and independence from location and time constraints. Smart-e-Moms may, therefore, be a promising tool to reduce the treatment gap for women with PPD [11, 12] and to provide empirically supported treatment for women who would not otherwise receive adequate care.

Our results will highlight whether a smartphone-based intervention for German-speaking women with PPD is utilized by affected women and has positive impacts on postpartum depressive symptoms, self-efficacy, and stress levels. Moreover, we will also assess the effects on mother-infant bonding, a factor often neglected in similar studies [18]. However, evidence suggests that not only symptoms of PPD negatively influence the child’s development, but the quality of mother–child interaction is crucial [57, 58]. Therefore, the effects of interventions for PPD should also include measures on maternal bonding to obtain possible indications of preventive effects on child development. Additionally, we will assess the short-term stability of any achieved effects. Moreover, by assessing a broad range of participant characteristics, covariates, and process-oriented variables (e.g., age, birth trauma, and therapeutic relationship), our findings will contribute to knowledge on women who benefit from a smartphone-based intervention for PPD and which underlying mechanisms mediate effects. To ensure adequate participant enrolment, we will use several recruitment channels. By recruiting via maternity hospitals, gynecologists, midwives, and pediatricians, the trial will be directly integrated into routine care. Additionally, we will use social media to inform potential participants about the trial.

This trial has several limitations: As the study compares the treatment group to an informed TAU, we must assume that the control group has more awareness and knowledge of their own vulnerability and may be more likely to actively seek conventional treatment than mothers from the general population. We are collecting data on healthcare utilization and adding them as moderators to assess their potential impact on our findings. Moreover, due to the short funding period, we are not assessing any long-term effects beyond 24 weeks, and we are not conducting a cost analysis.

Smart-e-Moms can become an important part of PPD care in German-speaking countries. At best, this intervention will not only contribute to maternal health but also enhance the opportunity for healthy child development. If Smart-e-Moms proves to be effective, the program represents a low-threshold, low-cost, and easily scalable intervention with a high potential for transfer to regular care.

## Trial status

This is the first version of the protocol (27/03/2024). Recruitment will start in August 2024, with the first enrolment planned for 15/08/2024. Recruitment will be completed 9 months after the first patient enrolment, around 30/04/2025.

## Data Availability

Part of the anonymized data is made publicly available on an internet database for open access documentation. No information (e.g., place of residence, date of birth) will be published that would allow the identification of individuals. Before selecting the data, consultation with the data protection officer will take place.

## Abbreviations

CBT: Cognitive-behavioral therapy
City BiTS: City Birth Trauma Scale
CONSORT: Consolidated Standards of Reporting Trials
CTS: Childhood Trauma Screener
EPDS: Edinburgh Postpartum Depression Scale
FIMPsy: Fragebogen zur Inanspruchnahme medizinischer und nicht medizinischer Versorgungsleistungen bei psychischen Erkrankungen
FU: Freie Universität Berlin
GBA: Gemeinsamer Bundesausschuss
GSE: General Self-Efficacy Scale
IBI: Internet-based intervention
ITT: Intention-to-treat
OSF: Open Science Framework
PATHEV: Patient Questionnaire on Therapy Expectation and Evaluation
PBQ-16: Parental Bonding Questionnaire
PPD: Postpartum depression
RCT: Randomized controlled trial
SPIRIT: Standard Protocol Items: Recommendations for Interventional Trials
TAU: Treatment as usual
U2: Second examination after birth
uMARS: User Version of the Mobile Application Rating Scale
UKE: Universitätsklinikum Hamburg-Eppendorf
WAI-SR: Working Alliance Inventory—short revised
